# Supplementary figures and images for: Computational analysis of missense filamin-A variants, including the novel p.Arg484Gln variant of two brothers with periventricular nodular heterotopia
Source: PLoS One. 2022 May 25;17(5):e0265400. doi: 10.1371/journal.pone.0265400 (PMC9132340; doi:10.1371/journal.pone.0265400)

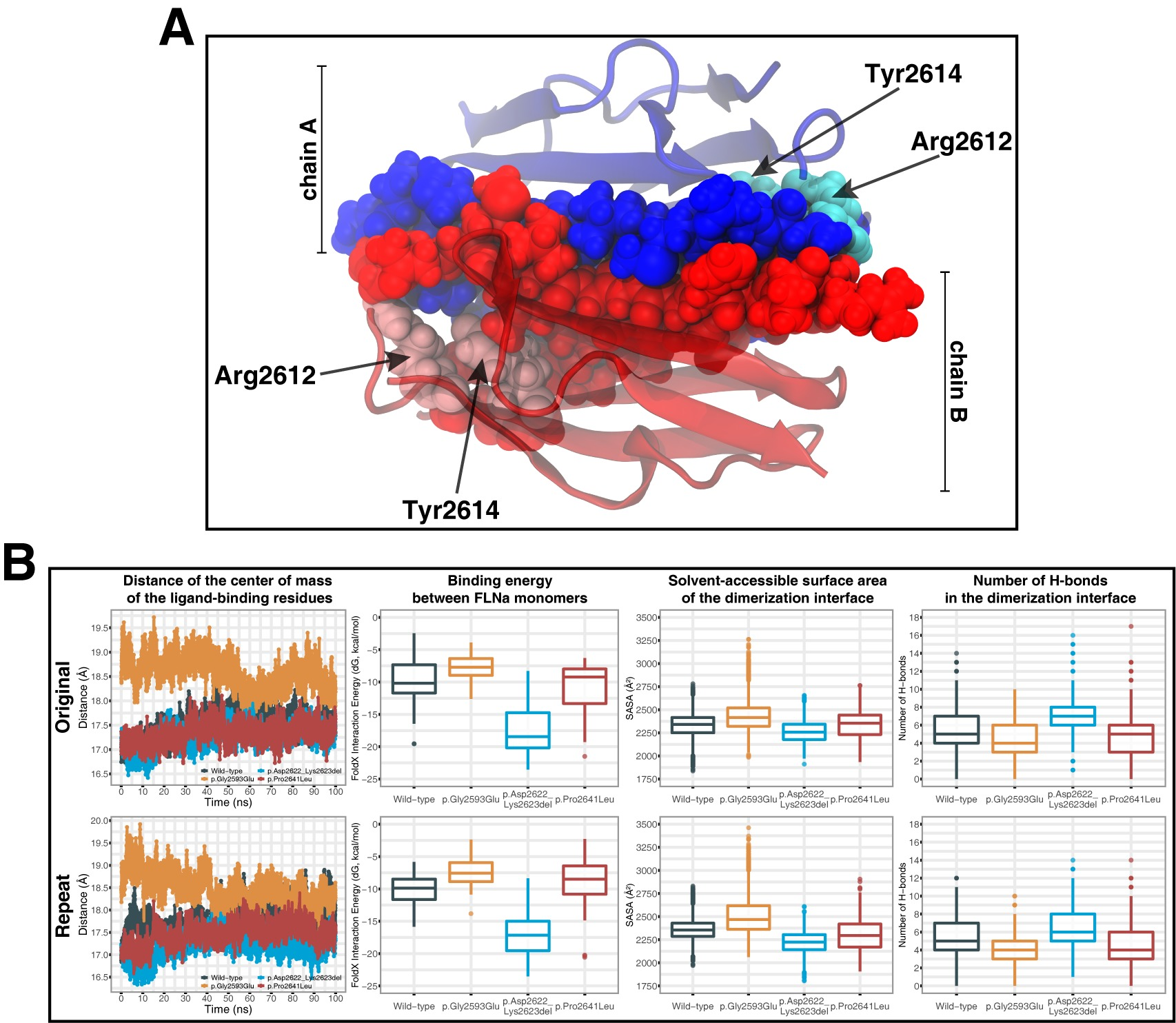

Supplement: S1 Fig — (A) Visualization of the dimerization interface in IgFLNa24 of filamin-A (FLNa), including the residues Arg2612 and Tyr2614. (B) Distance between the IgFLNa24 monomers through the time, binding energy distributions between the monomers, distributions of number of H-bonds and solvent-accessible surface area (SASA) in the dimerization interface during the molecular dynamics simulations. (TIF) [file pone.0265400.s001.tif]

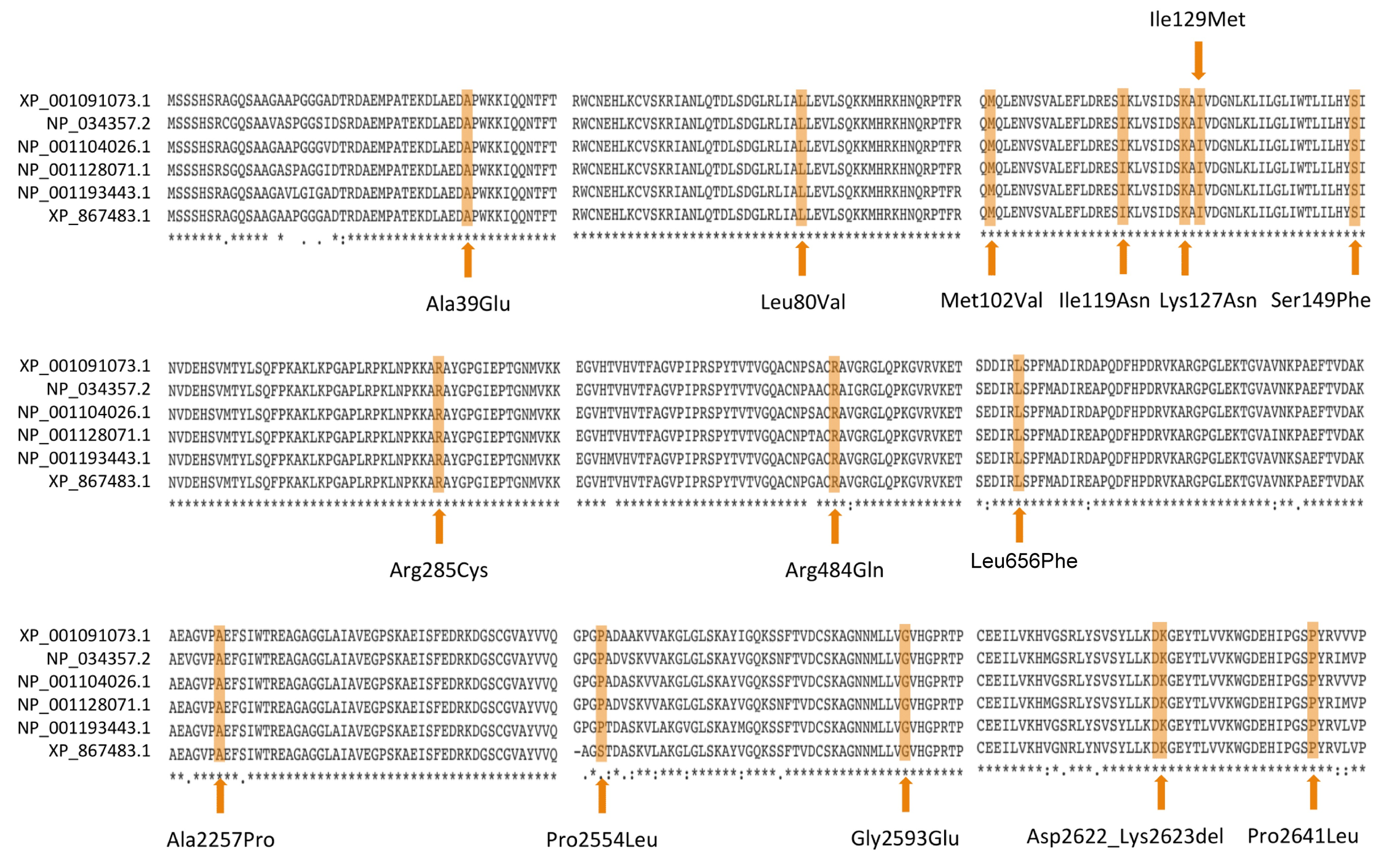

Supplement: S2 Fig — The filamin-A orthologs from human (Homo sapiens, NP_001104026.1), rhesus macaque (Macaca mulatta, XP_001091073.1), dog (Canis lupus familiaris, XP_867483.1), bovine (Bos taurus, NP_001193443.1), mouse (Mus musculus, NP_034357.2) and brown rat (Rattus norvegicus, NP_001128071.1) were used. (TIF) [file pone.0265400.s002.tif]

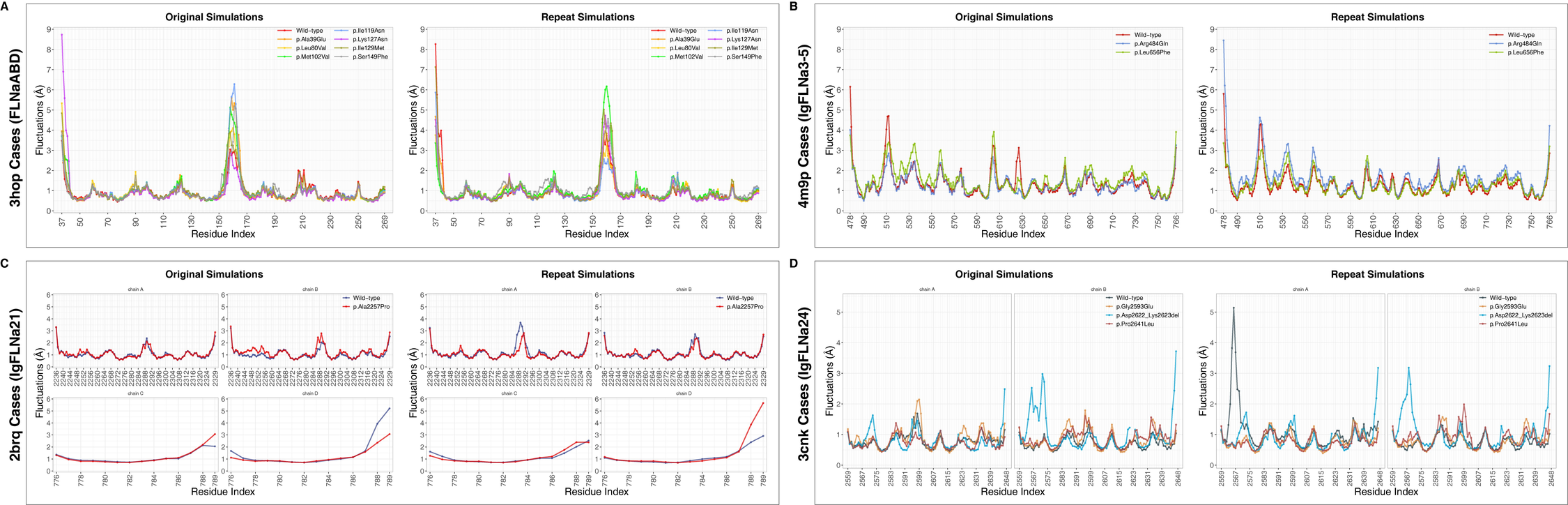

Supplement: S3 Fig — (TIF) [file pone.0265400.s003.tif]

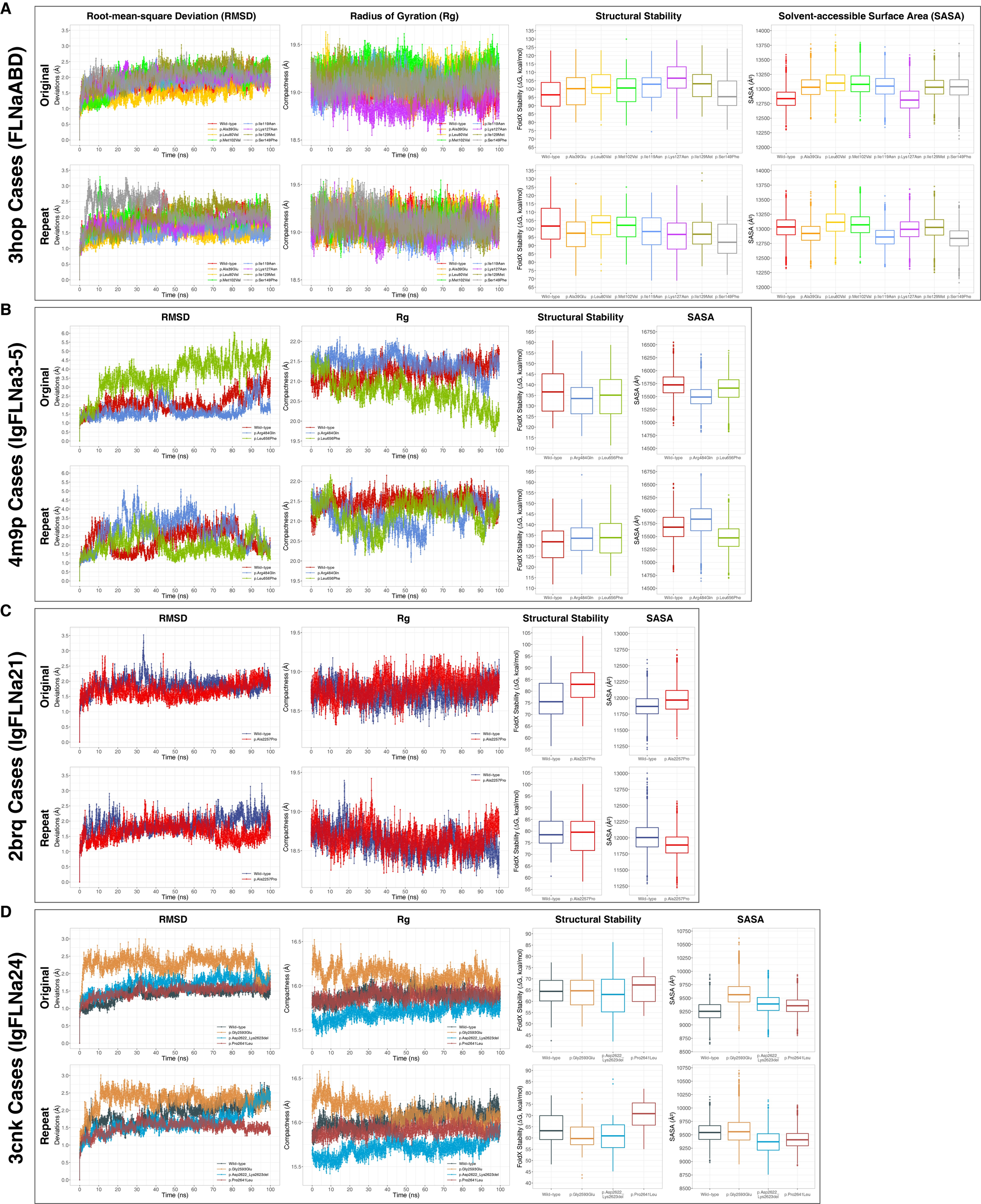

Supplement: S4 Fig — The results are plotted as against the time (RMSD and Rg) or as the distribution of collected values during the MD simulations (stability and SASA). (TIF) [file pone.0265400.s004.tif]

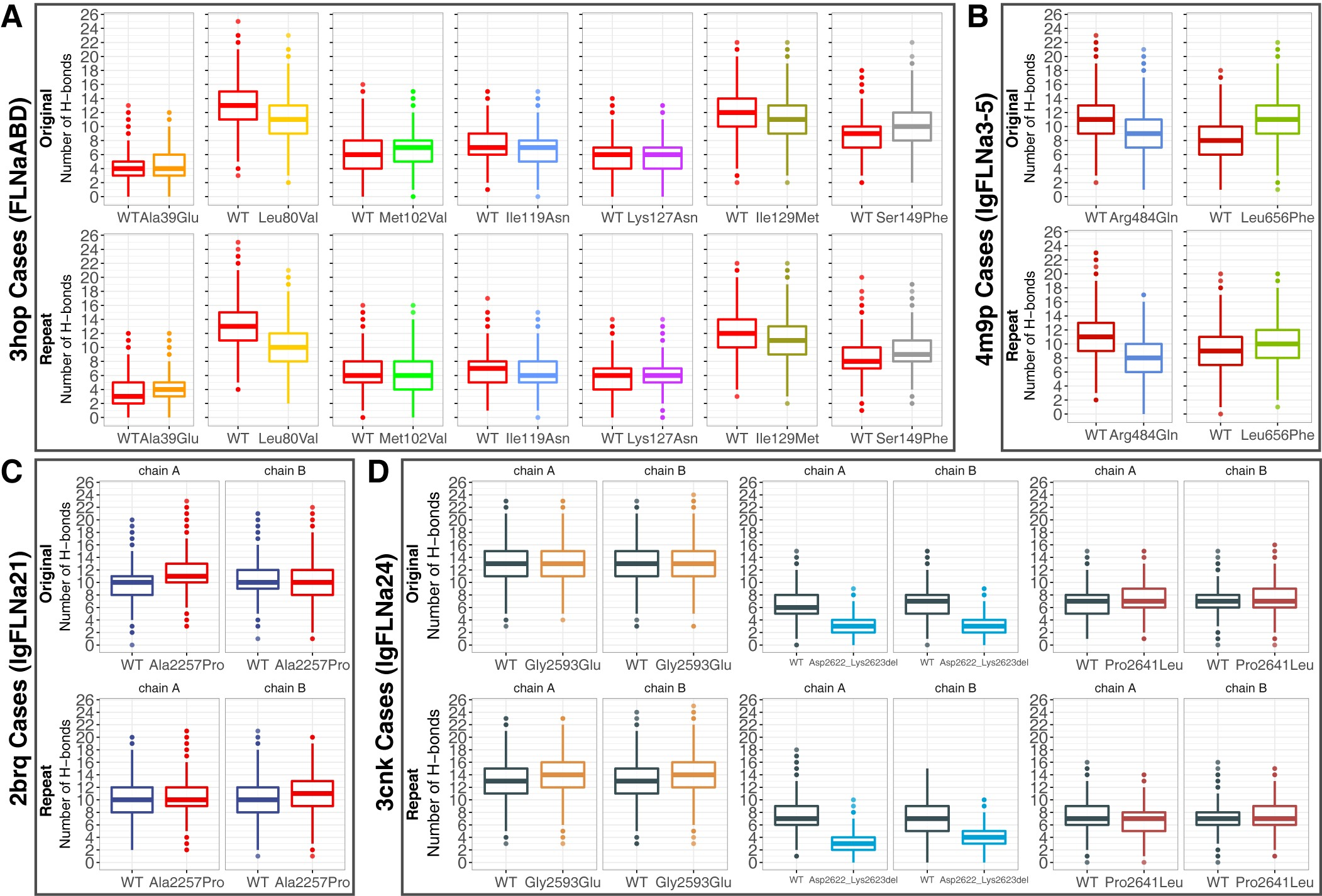

Supplement: S5 Fig — (TIF) [file pone.0265400.s005.tif]

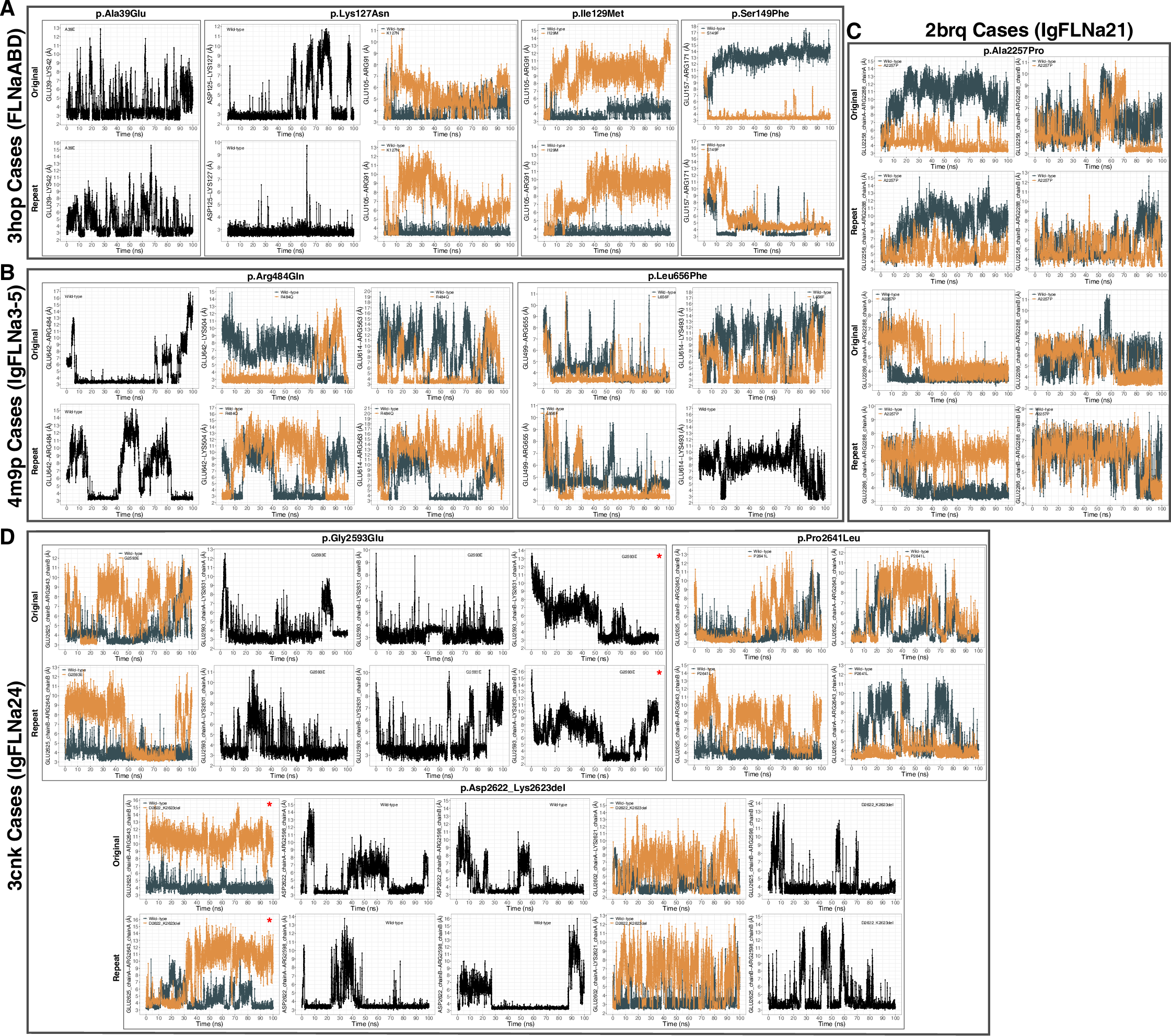

Supplement: S6 Fig — (TIF) [file pone.0265400.s006.tif]
