# Supplementary material for: Computational analysis of missense filamin-A variants, including the novel p.Arg484Gln variant of two brothers with periventricular nodular heterotopia
Source: PLoS One. 2022 May 25;17(5):e0265400. doi: 10.1371/journal.pone.0265400 (PMC9132340; doi:10.1371/journal.pone.0265400)
Supplement: S1 Table — †EMD is the gene encoding Emerin protein. (DOCX) [file pone.0265400.s007.docx]

**S1 Table. All periventricular nodular heterotopia-associated filamin-A variants reported in the liveborn males.**

| **Number of patients** | **Amino acid alteration** | **Nucleotide alteration** | **Type of mutation** | **Ref** |
| --- | --- | --- | --- | --- |
| 1 | p.Ala39Glu | c.116C>A | Missense | Fergelot *et al*, 2012 |
| 1 | p.Leu80Val | c.238C>G | Missense | Cannaerts *et al.,* 2018 |
| 1 | p.Met102Val | c.304A>G | Missense | Guerrini *et al.,* 2004 |
| 1 | p.Ile119Asn | c.356T>A | Missense | Fergelot *et al*, 2012 |
| 1 | p.Lys127Asn | c.381G>C | Missense | Reinstein *et al.,* 2013 |
| 1 | p.Ile129Met | c.387C>G | Missense | Reinstein *et al.,* 2013 |
| 1 | p.Ser149Phe | c.446C>T | Missense | Guerrini *et al.,* 2004 |
| 1 | p.Arg285Cys | c.853C>T | Missense | Reinstein *et al.,* 2013 |
| 1 | p.Arg285Cys | c.853C>T | Missense | Jenkins *et al.,* 2018 |
| 1 | p.Lys331Ter | c.994delG | Nonsense | Masurel-Paulet *et al.*, 2011 |
| 1 | p.Lys355Lys, potential to disrupt splicing | c.1065G>A | Synonymous, Splice site | Jenkins *et al.,* 2018 |
| 1 | p.Gly475Ter | c.1425C>A | Nonsense | Liu *et al*., 2017 |
| **2 (Brothers)** | **p.Arg484Gln** | **c.1451G>A** | **Missense** | **This study** |
| 1 | Truncation at 574 upon  exon 12 skipping | A>G, 2 bp upstream of exon 12 | Splice Site | Parrini *et al.*, 2004 |
| 1 | Truncation at 574 upon  exon 12 skipping | A>G, 2 bp upstream of exon 12 | Splice site | Guerrini *et al.,* 2004 |
| 1 | p.Gly640Gly,  premature truncation  at 681 (predicted) | c.1923C>T | Synonymous,  Splice Site | Hehr *et al*., 2006 |
| 1 | p.Leu656Phe | c.1966C > T | Missense | Sheen *et al*., 2001 |
| 1 | p.Arg760fs*25 | c.2280+389T > A | Frameshift | Jenkins *et al.,* 2018 |
| 1 | Likely to destabilize the  entire mRNA and  truncate any protein  product | AGGAGGTG del, 4 bp downstream of exon 25 | Splice site | Guerrini *et al.,* 2004 |
| 1 | Duplication of IgFLNa13  (and a duplication in Emerin protein) | g.(EMD ex1)_(FLNAex26)dup | Partial duplication | Jenkins *et al.,* 2018 |
| 1 | p.Ala1833_Ser1835delinsAsp | c.5498_5504delCACCCACinsAC | Deletion  +insertion | Reinstein *et al.,* 2013 |
| 1 | p.Gly1896Arg, abolish correct splicing of intron 35 (predicted) | c.5686G>A | Missense, Splice site | Kasper *et al.*, 2013 |
| 2 | p.(Glu2142AlafsTer22) | c.6425_6428delAGAG | Frameshift | Oda *et al*., 2015 |
| 1 | p.Ala2257Pro | c.6769G>C | Missense | Hommel *et al.,* 2016 |
| 1 | p.Tyr2305Ter | c.6915C >G | Nonsense | Sheen *et al*., 2001 |
| 1 | p.Gln2341Ter | c.7021C>T | Nonsense | Kapur *et al.,* 2010 |
| 1 | p.Ser2352Ter | c.7055_7070delCTTTTGCAGTCAGCCT | Nonsense | Lange *et al.*, 2015 |
| 1 | p.Val2464Alafs*5 | c.7391_7403del | Frameshift | Pelizzo *et al.*, 2019 |
| 1 | p.Pro2554Leu | c.7661C>T | Missense | Liu *et al*., 2017 |
| 2 | p.Gly2593Glu | c.7778G>A | Missense | Kogelenberg *et al.,* 2015 |
| 2 | p.Asp2622_Lys2623del | c.7865_7870del | Inframe deletion | Parrini *et al.*, 2011 |
| 1 | p.Asp2634Thrfs*71 | c.7900delG | Frameshift | Kogelenberg *et al.,* 2015 |
| 2 (Dizygotic twin boys) | p.Pro2641Leu | c.7922C>T | Missense | Gerard-Blanluet *et al*., 2006 |
| 3 | p.(*2648Serext*100) | c.7941_7942delCT | No-stop, Frameshift | Oegema *et al*., 2013 |

^†^*EMD* is the gene encoding Emerin protein.
