# Supplementary material for: Computational analysis of missense filamin-A variants, including the novel p.Arg484Gln variant of two brothers with periventricular nodular heterotopia
Source: PLoS One. 2022 May 25;17(5):e0265400. doi: 10.1371/journal.pone.0265400 (PMC9132340; doi:10.1371/journal.pone.0265400)
Supplement: S2 Table — (DOCX) [file pone.0265400.s008.docx]

**S2 Table. Classes of evidence in our variant prioritization strategy.**

| **Evidence Level** | **Evidence** | **Aim and Examples** |
| --- | --- | --- |
| **Gene Level** | Genetic | ***the gene shows statistically low number of variants*** |
|  |  | Exome-Aggregation-Consortium-Database (ExAC)  Genome-Aggregation-Database (GnomAD)  Genic-Intolerance |
|  | Experimental | ***Model systems: Animal models with mutated/knock-out gene present a phenotype that has overlaps with the human disease*** |
|  |  | Mouse Genome Informatics (MGI) |
|  |  | ***Protein Interactions: The product of the gene interacts with proteins which found to be related with the disease of interest*** |
|  |  | Protein Interactions: STRING |
|  | Literature | ***Biochemical function: The product of the gene has***  ***a function consistent with the phenotype*** |
|  |  | Deep literature search |
| **Variant Level** | Genetic | ***the variant is found in databases with a very low frequency or not found in any databases of healthy population cohorts*** |
|  |  | ***the variant is co-inherited with the disease in affected families*** |
|  |  | PopFreq  Exome-Aggregation-Consortium Database (ExAC)  Genome-Aggregation-Database (GnomAD) |
|  | Informatic | ***Conservation: the variant show evolutionary conservation*** |
|  |  | ***Predicted affect on function: the variant is found on the gene predicted to cause functional effect*** |
|  |  | Combined-Annotation–Dependent-Depletion (CADD)  Rare-Exome-Variant-Ensemble-Learner (REVEL)  The Mendelian-Clinically-Applicable Pathogenicity (M-CAP)  PrimatAI  Human Splicing Finder (HSF)  NetGene2 Server  Berkeley-Drosophila-Genome-Project-Splice Site Prediction by Neural-Network  Oriel-SpliceView  SIFT  PolyPhen2  MutationTaster  Homozygosity Mapper  BCFtools/ROH |
|  | Simulation | ***Comperative studies on the wild-type and mutated protein structures and dynamics*** |
|  |  | Elastic Network Models (ENM)  Molecular Dynamics (MD) Simulations |
|  | Literature | ***Whether the variation is located at the functional domains/motifs or the mutational hotspots of the protein*** |
|  |  | Deep literature search |
